# Therapeutics: Its Principles and Practice

**Published:** 1888-10

**Authors:** 


**Therapeutics: Its Principles and Practice.** A work on Medical Agencies, Drugs and Poisons, with especial reference to the relations between Physiology and Clinical Medicine. By H. C. Wood, M. D., LL.D., Professor of Materia Medica and Therapeutics, and Clinical Professor of Diseases of the Nervous System, in the University of Pennsylvania. The seventh edition of a treatise on Therapeutics, rearranged, rewritten and enlarged; 8vo, pp. 908; extra cloth. Philadelphia: J. B. Lippincott & Co. Price, $6.00. From Otto Ulbrich, bookseller, Buffalo.

A new edition of Dr. Wood's work always marks an advance in our knowledge of the treatment of disease. This is even more true of this than of previous editions. It is practically a new book, and contains in detail or by reference all that is now known on the subjects of which it treats. In these days of rapid advance in the treatment of disease, such a book is a necessity.

There is nothing, so far as we know, that quite takes its place. In comparing it with the last edition, we notice, in the first place, that the consideration of the remedial measures used in the treatment of disease, other than drugs, receives enlarged and extended treatment. This subject forms Part I. of the book, and discusses, among certain miscellaneous measures, *massage, metalotherapy, the feeding of the sick, and the dietetic and general treatment of underlying bodily constitutional states or diatheses, such as exhaustion, obesity, and lithiasis.* Much could be said in praise of the way these subjects are handled, and we anticipate that both teacher and student will find much of profit in the careful reading of this part of the work. Part II., devoted to the study of drugs, has been entirely rearranged according to a new classification. While this is too lengthy to quote completely, we feel sure this classification approaches nearer to a scientific standard than the one in the previous editions. It will be impossible for some time to have a satisfactory classification of drugs, but it is certain that we are nearer it to-day than heretofore. These are the principal new features of the work, though to appreciate them fully they should be carefully studied. We have a criticism to offer in regard to the arrangement of the references. The incorporation of them in the body of the text is a mistake. They should be collected together at the foot of each page, and not annoy the reader with their constant and voluminous interpolation. We have had many complaints from students upon this point. We appreciate the value of these references, but we protest against the necessity of either reading them or avoiding them. The latter is a difficult thing to do, and they interrupt seriously the study of the articles. We trust the time will come when the author will change them as suggested, and he will then have not only a most valued treatise but the best text-book on Therapeutics in the language.

